# Supplementary material for: Prophylactic Treatment with Baloxavir Protects Mice from Lethal Infection with Influenza A and B Viruses
Source: Viruses. 2023 Nov 16;15(11):2264. doi: 10.3390/v15112264 (PMC10675732; doi:10.3390/v15112264)
Supplement: Supplementary file 1 [file viruses-15-02264-s001.zip › viruses-2670110-supplementary.pdf]

Supplementary materials

Table S1. Plasma concentration of baloxavir acid after a single subcutaneous administration to mice.

Time	Plasma concentration (ng/mL)											
	1.6 mg/kg			3.2 mg/kg			6.4 mg/kg			10 mg/kg		
(hr)	Mean	±	SD	Mean	±	SD	Mean	±	SD	Mean	±	SD
2	N.T.			N.T.			N.T.			44.0		15.9
4	N.T.			N.T.			N.T.			47.2		6.8
8	N.T.			N.T.			N.T.			34.4		4.9
24	N.T.			N.T.			N.T.			26.1		1.3
48	3.39	±	0.57	5.45	±	0.65	11.2	±	0.8	17.8	±	2.8
72	2.25	±	0.59	3.58	±	1.19	8.70	±	1.26	N.T.		
96	0.883	±	0.542	2.35	±	0.32	6.40	±	1.70	N.T.		
120	0.444	±	0.183	2.07	±	0.58	4.80	±	0.45	6.79	±	0.89
168	N.T.			N.T.			N.T.			6.51	±	0.40
192	0.0495	±	0.0990	0.986	±	0.240	2.83	±	0.41	N.T.		
216	N.T.			N.T.			N.T.			5.97		0.72

The lower limit of quantification (BLQ) was < 0.1 ng/mL. The plasma concentrations for treatment with 10 mg of baloxavir are from another study [15]. The plasma concentration-time profile of baloxavir acid is depicted in Figure 1.

Table S2. Comparison of virus titres between vehicle and baloxavir acid treatments for overall time.

Virus	Dose (mg/kg)	Dose timing (hours before infection)	Difference of LS mean ± SE ($\log_{10}$ $TCID_{50}$, versus vehicle group)	P-value (versus vehicle group)
A/PR/8/34	1.6	96	-0.734 ± 0.119	<0.0001
	3.2	72	-2.270 ± 0.118	<0.0001
B/Hong Kong/5/72	3.2	72	-0.513 ± 0.080	<0.0001
	6.4	48	-1.154 ± 0.080	<0.0001

Viral titres in supernatants of lung homogenates were determined (n = 10 per group) on days 1, 2, 4, 6, 8 and 10 after infection by standard $TCID_{50}$ assay. For pairwise comparison of virus titres in lung tissues among groups for the overall time, the two-way analysis of variance model was applied. Observations on days 8 and 10 after infection were excluded from the analysis set of the ANOVA model, because the virus titre data were not available for most of the mice in the vehicle group due to death.

Table S3. Emergence of influenza A variants with amino acid substitutions during prophylactic treatment with baloxavir acid in mice.

Dose (mg/kg)	Dose timing (hours before infection)	Days post-infection	Substitution in PA
10	24	-	none
3.2	72	2	I38T (n=1)
		4	A36T (n=2)
1.6	96	2	A36A/T (n=1) [a]

Sequence analysis of viral RNA was performed using lung homogenates collected in the A/PR/8/34 (H1N1) infection study described above. Viral RNA was isolated from lung homogenates using the Quick-RNA Viral 96 Kit (Zymo Research). The N-terminal side of the PA region was amplified by RT-PCR. The primer sequences used are available upon request. Sanger sequence analysis was performed by Eurofins Genomics K.K. (Japan). [a] Detected as double peaks for A36A (wild) and A36T substitution.

Table S4. Susceptibility of the variants to baloxavir acid

	Baloxavir acid		Favipiravir	
	EC_{50} (nM)	Fold change	EC_{50} (μM)	Fold change
Parent virus	1.69 ± 0.57	N/A	22.3 ± 1.01	N/A
A36T virus	2.59 ± 1.33	1.53	19.1 ± 4.87	0.86

In order to investigate the impact of the A36T substitution, drug sensitivity testing was conducted by means of a plaque reduction assay with baloxavir acid, following plaque purification from the lung homogenate. Fold change was calculated by dividing the EC_{50} value by that of the parent virus. N/A = not applicable.

Figure S1. Plasma concentration of baloxavir acid after a single subcutaneous administration in mice in comparison with simulated PK profiles in humans.

(A) 10 mg/kg dosing 24 hours before infection

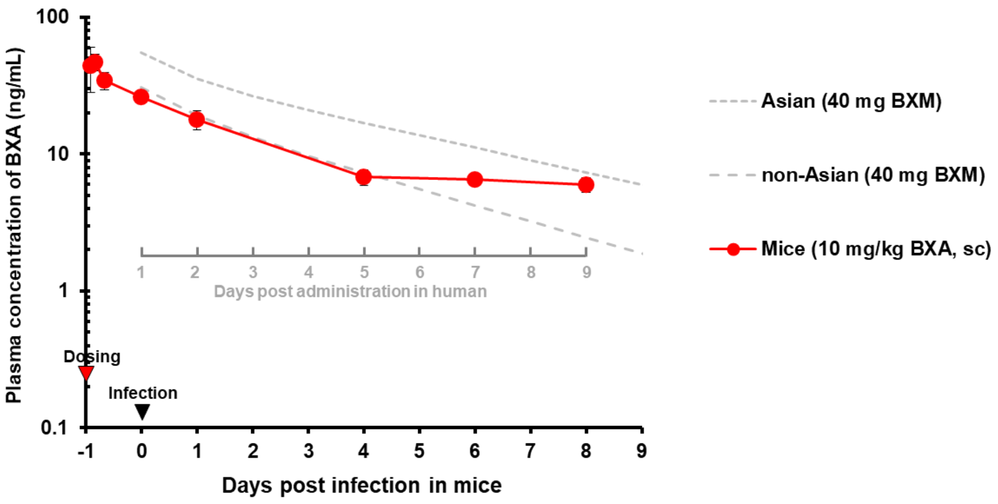

(B) 6.4 mg/kg dosing 48 hours before infection

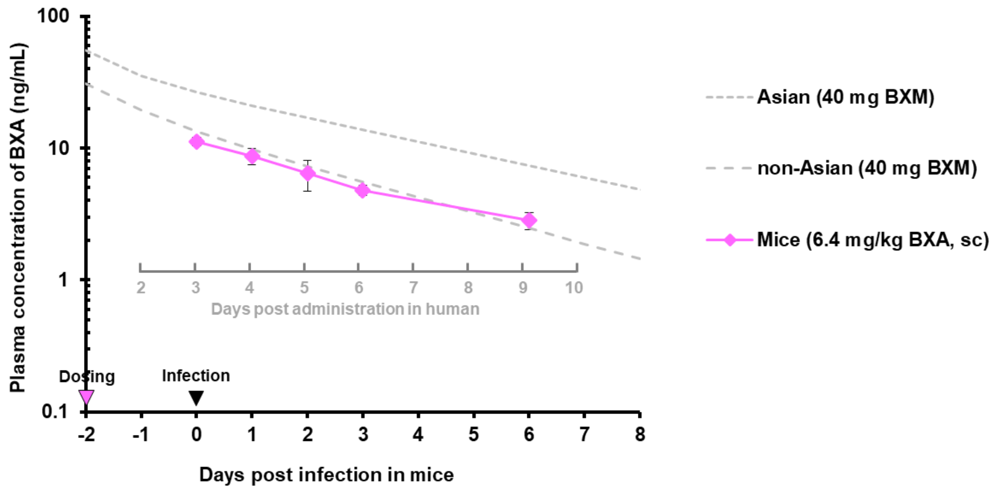

(C) 3.2 mg/kg dosing 72 hours before infection

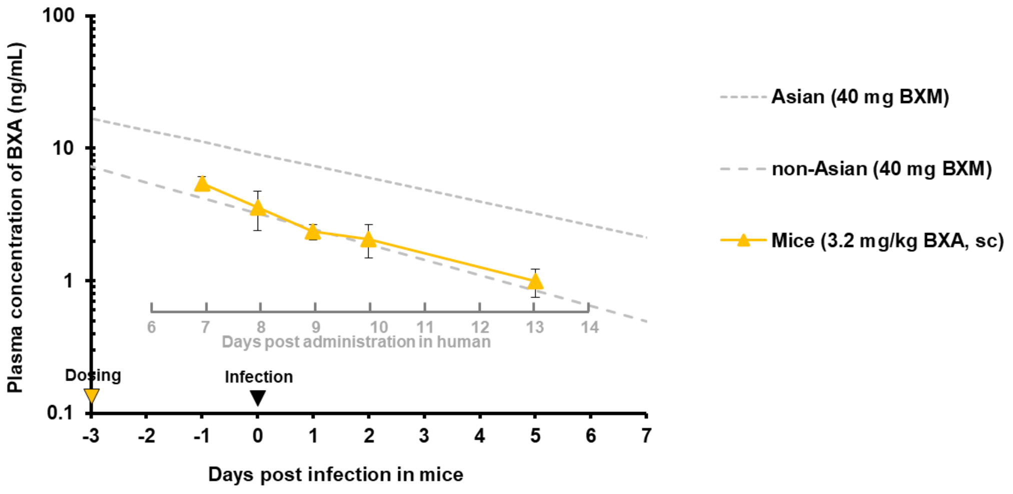

(D) 1.6 mg/kg dosing 96 hours before infection

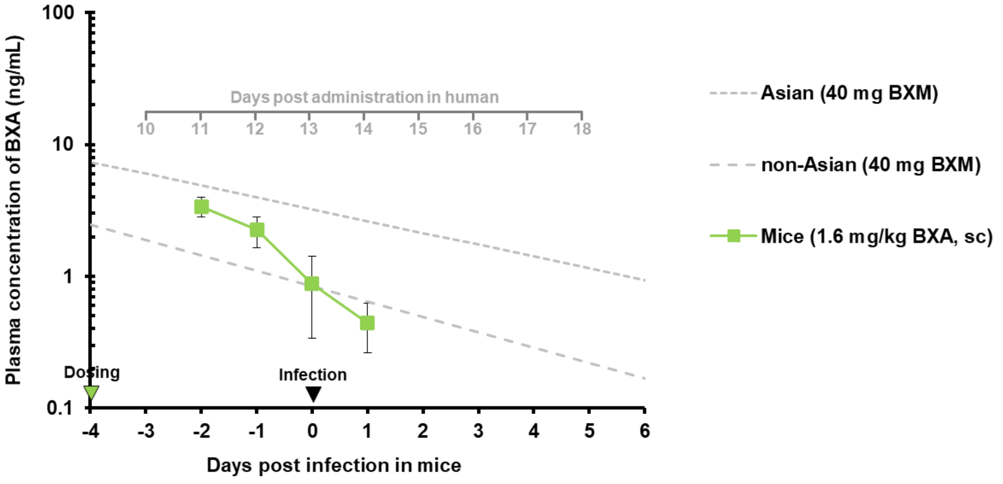

Superposition placed the PK profile for baloxavir acid (BXA) of mice over that of humans administered 40 mg baloxavir marboxil (BXM). The grey x-axis indicates the days post administration in humans. Dashed lines represent Asian and non-Asian PK profiles derived from population pharmacokinetics and exposure-response analyses in adults and adolescents [16].
